# Moving interdisciplinary science forward: integrating participatory modelling with mathematical modelling of zoonotic disease in Africa

**DOI:** 10.1186/s40249-016-0110-4

**Published:** 2016-02-25

**Authors:** Catherine Grant, Giovanni Lo Iacono, Vupenyu Dzingirai, Bernard Bett, Thomas R. A. Winnebah, Peter M. Atkinson

**Affiliations:** ESRC Social, Technological and Environmental Pathways to Sustainability (STEPS) Centre, Institute of Development Studies, Library Road, Falmer, Brighton UK; Geography and Environment, University of Southampton, Highfield, Southampton SO17 1BJ UK; Lancaster University, Lancaster, UK; University of Utrecht, Utrecht, UK; Queen’s University Belfast, Belfast, UK; Department of Veterinary Medicine, Disease Dynamics Unit, University of Cambridge, Cambridge, UK; Centre for Applied Social Sciences Trust, 5 Aberdeen Road, P O Box A1333, Avondale, Harare Zimbabwe; International Livestock Research Institute, Naivasha Road, Kabete, Nairobi Kenya; Institute of Geography and Development Studies, School of Environmental Sciences, Njala University, 17, Henry Street, Freetown, Sierra Leone

**Keywords:** Africa, Zoonoses, Modelling, Interdisciplinary, One health, Participatory, Disease

## Abstract

This review outlines the benefits of using multiple approaches to improve model design and facilitate multidisciplinary research into infectious diseases, as well as showing and proposing practical examples of effective integration. It looks particularly at the benefits of using participatory research in conjunction with traditional modelling methods to potentially improve disease research, control and management. Integrated approaches can lead to more realistic mathematical models which in turn can assist with making policy decisions that reduce disease and benefit local people. The emergence, risk, spread and control of diseases are affected by many complex bio-physical, environmental and socio-economic factors. These include climate and environmental change, land-use variation, changes in population and people’s behaviour. The evidence base for this scoping review comes from the work of a consortium, with the aim of integrating modelling approaches traditionally used in epidemiological, ecological and development research. A total of five examples of the impacts of participatory research on the choice of model structure are presented. Example 1 focused on using participatory research as a tool to structure a model. Example 2 looks at identifying the most relevant parameters of the system. Example 3 concentrates on identifying the most relevant regime of the system (e.g., temporal stability or otherwise), Example 4 examines the feedbacks from mathematical models to guide participatory research and Example 5 goes beyond the so-far described two-way interplay between participatory and mathematical approaches to look at the integration of multiple methods and frameworks. This scoping review describes examples of best practice in the use of participatory methods, illustrating their potential to overcome disciplinary hurdles and promote multidisciplinary collaboration, with the aim of making models and their predictions more useful for decision-making and policy formulation.

**Electronic supplementary material:**

The online version of this article (doi:10.1186/s40249-016-0110-4) contains supplementary material, which is available to authorized users.

## Multilingual abstracts

Please see Additional file 1 for translations of the abstract into the six official working languages of the United Nations.

## Introduction

This paper focuses on interdisciplinary modelling for zoonotic diseases from a One Health perspective. This is a belief that the emergence, risk, spread and control of diseases are affected by many complex bio-physical, environmental and socio-economic factors [1]. There are many benefits of using interdisciplinary research methods, this paper concentrates on the use of participatory modelling and local perspectives to enhance mathematical modelling.

Models are useful in disease research as they can provide characterisations and predictions to advance knowledge, and evidence to inform management decisions and policy formation [2–5]. However models also reproduce and are constructed based on their different disciplinary boundaries [6], despite this they are seen as valid representations of the world associated with objectivity and rationality [7]. They are limited by the data available and are influenced by perspective and even the political and funding arena in which particular research may be occurring. In addition, flaws due to an uncritical use of mathematical modelling in life science has also been pointed out [8]. The persuasiveness of numerical data and ‘quantitative authority’ can obscure the benefits of multidisciplinary collaboration, and the use of participatory research, which can make models and their predictions more useful for decision-making and policy formulation [6, 9].

The benefits of participatory research are important to take into account as many projects are designed by epidemiologists, veterinarians or entomologists with little, if any, input from social scientists [10, 11]. Additionally, debates related to infectious disease are often centred on professional and bureaucratic interests, while those actually living with disease in poor and marginal areas have little or no say in the particular interests prioritised, or how they are studied. Commonly, local people and their leadership are considered insufficiently knowledgeable to engage in scientific debates on the issues that might affect them [11].

Moreover, local people can provide useful information, including data unavailable anywhere else, and this is being increasingly recognised [12]. For example, a recent paper on modelling Lassa Fever stated; ‘participatory modelling and ethnographic research would be an invaluable tool to assess the variety of practices and settings in which people come into contact with each other’s urine and other body fluids, perceptions of risk, and approaches to hygiene’ [2]. However as Leach and Scoones [6] pointed out there is a lot of research which could benefit from including a participatory approach e.g. [13, 14]. With increased collaboration, participatory research can be further utilised for effective integration of mathematical modelling, fieldwork and data. Likewise, there is much to be gained from the potential benefits to programmatic work of increased dissemination and knowledge transfer to communities in a suitable format. The problem is, of course, complex and it will require ‘reflexivity, humility and interaction amongst modellers, policymakers and those living with diseases’ in order to be implemented successfully [6, 15].

Controlling zoonotic disease outbreaks has become ever more important; it has been estimated that since 1940, about 60 % of the emerging infectious diseases affecting humans globally, but mainly in developing countries, have originated from animals, both domestic and wild [16]. Additionally, One Health research potentially offers great benefits compared to traditional, single-disciplinary research, with participatory research adding people’s own cultural logic and potentially alternative, policy-relevant perspectives compatible with local cultural values and livelihood priorities.

Research which seeks to integrate different methodological perspectives, has shown that participatory research can add context, depth, legitimacy and accuracy to the modelling approaches of other disciplines, and it can be particularly useful when investigating climate and anthropogenic environmental change as drivers of disease emergence. This paper discusses the potential value of participatory approaches for process-based modelling and the models are described as part of the case study examples and in detail in previous papers [2].

## Review

### Dynamic drivers of disease

The evidence for this paper is based on One Health research on zoonoses which employed five methodological themes across five African country case studies over a two year fieldwork period in which four zoonotic diseases were considered. The case study diseases and countries focused on are described in Table 1. The consortium which conducted this research has an ethics policy in place, an appointed country lead for ethics in each of the five countries it is working in and the appropriate ethics approvals have been sought. The ethics committees which provided approval were: The Institutional Review Board of the Noguchi Memorial Institute of Medical Research, Ghana, African Medical and Research Foundation Ethics and Scientific Review Committee (AMREF-ESRC), Kenya, ERES Converge IRB, Zambia, The Animal Welfare and Ethical Review Body, University of Edinburgh for Zimbabwe and Sierra Leone Ethics and Scientific Review Committee (SLESRC), Sierra Leone.

**Table 1:** Case study diseases, dynamics and poverty impacts and some modelling issues

Case study disease	Key ecosystem-disease dynamics	Poverty and wellbeing impacts	Key modelling issues, most of which depend on human activities
Lassa fever in Sierra Leone. Lassa fever is a rodent-borne, viral haemorrhagic fever endemic in West Africa. The natural reservoir of Lassa is the ‘multimammate rat’ of the genus *Mastomys. Mastomys* often colonise houses, where Lassa may be transmitted to humans via contact with rodent excreta; human-to-human transmission is also possible.	Changing land use and settlement patterns increasing transmission from *Mastomys natalensis.*	Significant impacts in poor farming, peri-urban and mining settlements. High fatality rates, with pregnant women particularly vulnerable. Increasing exposure especially for poor people living in rapidly-growing crowded conditions with poorly-constructed houses. Estimates of 500,000 cases per year but significant under-diagnosis.	Proportion of transmission due to human-to-human routes not fully assessed, only recently theoretical estimation provided [2]. Unclear disease dynamics in the reservoir (*e.g.* potential vertical transmission, role and extent of immunity). Unclear ecology of the reservoir (e.g. dispersal patterns). Route of transmission still unclear. Apparent seasonality in disease incidence but of unclear origin. Most life history and contact parameters unknown.
Henipavirus in Ghana. Henipaviruses in the family *Paramyxoviridae* comprise Hendra virus and Nipah virus. Pteropid bats are reservoir hosts for Nipah virus. Both Hendra and Nipah virus are zoonotic, causing encephalitic disease in humans with case fatality rates of around 75 %. The virus can spill over via infected pigs or directly from bats into people. Human-to-human transmission of the virus, with fatal consequences, has been documented. There are no pteropid bats in mainland Africa, but the closely-related straw-coloured fruit bat (*Eidolon helvum*) is widespread and populous and henipaviruses are maintained within this species across its geographic range.	Agricultural land-use change affecting bat roosting and migration patterns; growing intensity of human interactions with bats including in urban areas.	Spillover identified already between bats and pigs in Malaysia and Singapore in 1998 and 1999; particular vulnerability of smallholder pig farmers, bushmeat hunters and traders, and urban poor exposed to bat roosts. Suspected mis-reporting in humans; symptoms (high fever and encephalitis) often attributed to malaria.	So far, no reported case of zoonotic spillover to humans in Ghana. Unclear disease dynamics in the reservoir. Unclear ecology of the reservoir (e.g. migratory patterns). Route of transmission still unclear. Life history and contact parameters unknown.
Rift Valley fever in Kenya. RVF has an interesting and imperfectly understood epidemiology. It is a zoonotic arbovirus affecting different species of livestock, wildlife and humans. It is transmitted mainly by different species of mosquitoes with different ecology and temporal patterns. The mosquito dynamics are driven essentially by the environmental dynamics of water bodies.	Climate-driven and irrigation/standing water-driven dynamics linking wildlife, livestock and human populations in pastoral areas.	Cyclical outbreaks with high impact including effects on human health, and disruption to livestock trade, with massive livelihood impact on often very poor populations.	Many hosts affected by the disease with different degree of susceptibility which is only partially known. Potential existence of wildlife reservoir. Uncertainty in the role of *Aedes* mosquitoes in transmitting the disease to offspring. Limited information on mosquitoes feeding patterns. Lack of detailed information on irrigation patterns and water body dynamics. Unclear ecology of the mosquitoes (e.g. abundance, seasonality) and how this is affected by water abundance.
Trypanosomiasis in Zambia and Zimbabwe. Trypanosomiasis is a widely studied disease vectored by the tsetse fly. The human form of the disease is called Human African Trypanosomiasis (HAT) or sleeping sickness, while the animal form, is called Animal African Trypanosomiasis (AAT) or *nagana*. In west Africa *T.b. Gambiense* affects humans only, while in eastern and southern Africa, the zoonotic *T.b. Rhodiesiense* affects both humans and animals. *T.b. brucei* rarely affects humans.	Circulates within wildlife populations via tsetse fly. Livestock can also act as a significant reservoir of disease. Interaction between humans, livestock and wildlife within ecosystems suitable for tsetse results in spillover.	Massive impacts on poor farming and livestock-raising communities, including human health impact estimated by the Global Burden of Disease studies at WHO at 8721 DALYs in Africa. Huge underestimation of human and poverty impacts.	Focus on trypanosomiasis in livestock, wildlife and humans. Aim to produce predictive distributions for tsetse presence/absence and for tsetse abundance within the study area. Aim to create an ABM simulation which incorporates individual agents allowing the ‘bottom-up’ modelling of a transmission network between humans, tsetse flies and animal agents over a detailed physical landscape. Areas of interest include the effects of seasonality, of changes in land cover and changes in population density.

The methodological themes used in the research, and providing the basis for this paper are macro-ecological modelling, process based modelling (based on a theoretical understanding of relevant bio-physical and social processes and includes many approaches), participatory methods, socio-economic methods (including systems dynamics representations) and political economy of knowledge analysis (Table 2).

**Table 2:** An outline of the two main methods

Method	Approach	Integration and pros and cons of this
Participatory research	A partnership approach to research that equitably involves, for example, community members, organizational representatives, and researchers in all aspects of the research process and in which all partners contribute expertise and share decision making and ownership (Israel et al. 1998)	Participatory research can (1) ensure culturally and logistically appropriate research, (2) enhance recruitment capacity, (3) generate professional capacity and competence in stakeholder groups, (4) result in productive conflicts followed by useful negotiation, (5) increase the quality of outputs and outcomes over time, (6) increase the sustainability of project goals beyond funded time frames and during gaps in external funding, and (7) create system changes and new unanticipated projects and activities. Negative examples illustrated why these outcomes were not a guaranteed product of PR partnerships but were contingent on key aspects of context (Jagosh et al. 2012) Participatory research, through community involvement can be helpful to other methods as it can: 1. Remove ignorance, provide new information 2. Confirm prior knowledge. 3. Remove irrelevant information. 4. Remind us of important information to include. 5. Remove erroneous information.
Process based models	Population models: a class of mathematical models which study the dynamics of populations such as changes in the size and age composition, and the processes affecting these changes. Agent Based Models: a class of mathematical models relying on computational resources to modelling systems composed of autonomous, interacting agents. “Agent-based modelling is a way to model the dynamics of complex systems and complex adaptive systems. Such systems often self-organize themselves and create emergent order. Agent-based models also include models of behaviour (human or otherwise) and are used to observe the collective effects of agent behaviours and interactions [70].	Compared to ABMs, population models are usually based on a parsimonious set of assumptions on the underlying mechanism. In general, this results in a more transparent interpretation of the predictions. They are often based on a set of differential equations (which can be stochastic) allowing well-established further analytical approaches (e.g. stability analysis). They tend to require little computational resources. In contrast, ABMs are *in-silico* experiments able to incorporate comprehensive and detailed biological, physical, environmental and behavioral factors. Compared to analytical approaches, they require a minor level of abstractions, which might be ad advantage for integration with participatory modeling.

### Participatory research

The participatory methods and techniques which were used are outlined in this section as well as the usefulness of participatory research for integrative modelling, first with a diagram illustrating how it can be utilised, followed by case study examples and the findings of this research and how it contributed to other modelling approaches. Participatory methods used in the case study countries included two types of participatory mapping, including landscape mapping and village epidemiological mapping. Landscape mapping focuses on a village territory in its entirety, with people mapping out and marking who goes where and why on a spatial map, including seasonal movements and sightings of animal disease vectors.

The above method contrasts with village epidemiological mapping, which maps a village area, showing which families live where, the relationships between people and animal movements, and possible risk areas for disease (e.g. toilets, garbage areas, wells). In addition, the map marks households where people have had fevers in order to visualise the patterns of disease, and explore the reasons why people believe that certain individuals have been sick, for example, for spatial, structural or personal reasons, with notes of this discussion taken. Epidemiological maps created at different time points can feed into (or at least help contextualise and explain) process-based epidemiological modelling, as well as link with serological and disease surveys. The maps, having a ‘social’ element, can also become the basis of wealth and poverty ranking exercises or structured surveys.

Other participatory methods undertaken included transect walks, livelihood profiles and participatory matrices. This involved walking through a particular landscape with villagers observing different land and ecosystem types, as marked on participatory maps. The observations are used as an opportunity for ‘on the spot’ conversations and gathering further information, for example, where animals or disease vectors are seen, where particular activities are carried out seasonally (seasonal calendars can also mark this), or where particular ecosystem services are used.

Livelihood profiles explore different sources of livelihood and income, how they are affected by access to, and control over, land and labour and the relationship between sources of livelihood ‘for the household’ and those controlled by individuals on their own account. Detailed guidance on these techniques have been produced. Participatory matrices were used to make a relative comparison between different options, and to make a detailed analysis of how much, why and how people make decisions. More detail can be found in published research e.g. [17].

To be able to replicate the above techniques, the importance of empowerment of local communities, training for those leading participatory research and a genuine understanding of communities are essential to ensure integration and representation of local knowledge and aspirations, and this depends on issues of ‘trust between holders of knowledge, process facilitators and the eventual users of knowledge’ [18]. A wide range of research on participatory mapping is available, allowing for understanding and replication of these techniques [8, 18, 19].

Additionally, assessing local people’s interests is critical to the continuation of activities beyond the active research period; it maximises the likelihood of making policy decisions that extend beyond the lifetime of projects and of providing incentives and guarantees that all, or a sufficient number of, people will contribute towards the creation of a public good [20, 21]. As we know from Ferguson’s *Anti Politics Machine* [22] and from the many transactional works by Barth, Bailey and others, some reluctance stems from fears of local populations changing the implementation of technologies in line with local objectives and understandings, away from scientific and technical advice. There appears to be little effort to address this shortfall [23]. The Communal Areas Management Programme for Indigenous Resources (CAMPFIRE), with its research groups interfacing with ecologists in the Zambezi Valley, represents a good example of a project focusing on creation of local awareness of studied issues [24]. However, even in this case efforts have generally stopped short of including local communities’ knowledge and expertise as evidence for decision-making and involving communities in policy decisions.

Support for interdisciplinary and integrative approaches may potentially help mitigate the impact of disease on people’s health, lives and livelihoods. They can also create legitimacy for science, and lay the foundation for the adoption of policy. Case studies, such as those presented in this paper, can help build One Health, giving detailed examination of the impact of participatory approaches on integrative modelling frameworks. This can also be transferred to help build much-needed effective practice as there is a need for well-functioning, integrated research, surveillance and practice in zoonotic diseases across the human and animal sectors.

### The use of participatory research to approach the optimal model

A mathematical system is a simplified representation of reality and anything outside the system is disregarded. This is inevitable to avoid the mathematics becoming intractable. However, participatory research can be combined with theoretical models to complement, and in some cases add, realism and accuracy to mathematical models.

A fundamental way in which participatory approaches can aid mathematical modelling is by helping to define the appropriate model and its structure. By this, we mean the choice of modelling approach (e.g. a continuous population model *vs* ABM [25–27]), model specification (e.g. its inputs and the functional form of their distribution), and the domain of interest (e.g. relevant space of parameters). Meaningful interpretation of the findings of the model is another area in which participatory research can be highly valuable.

**Fig. 1:**
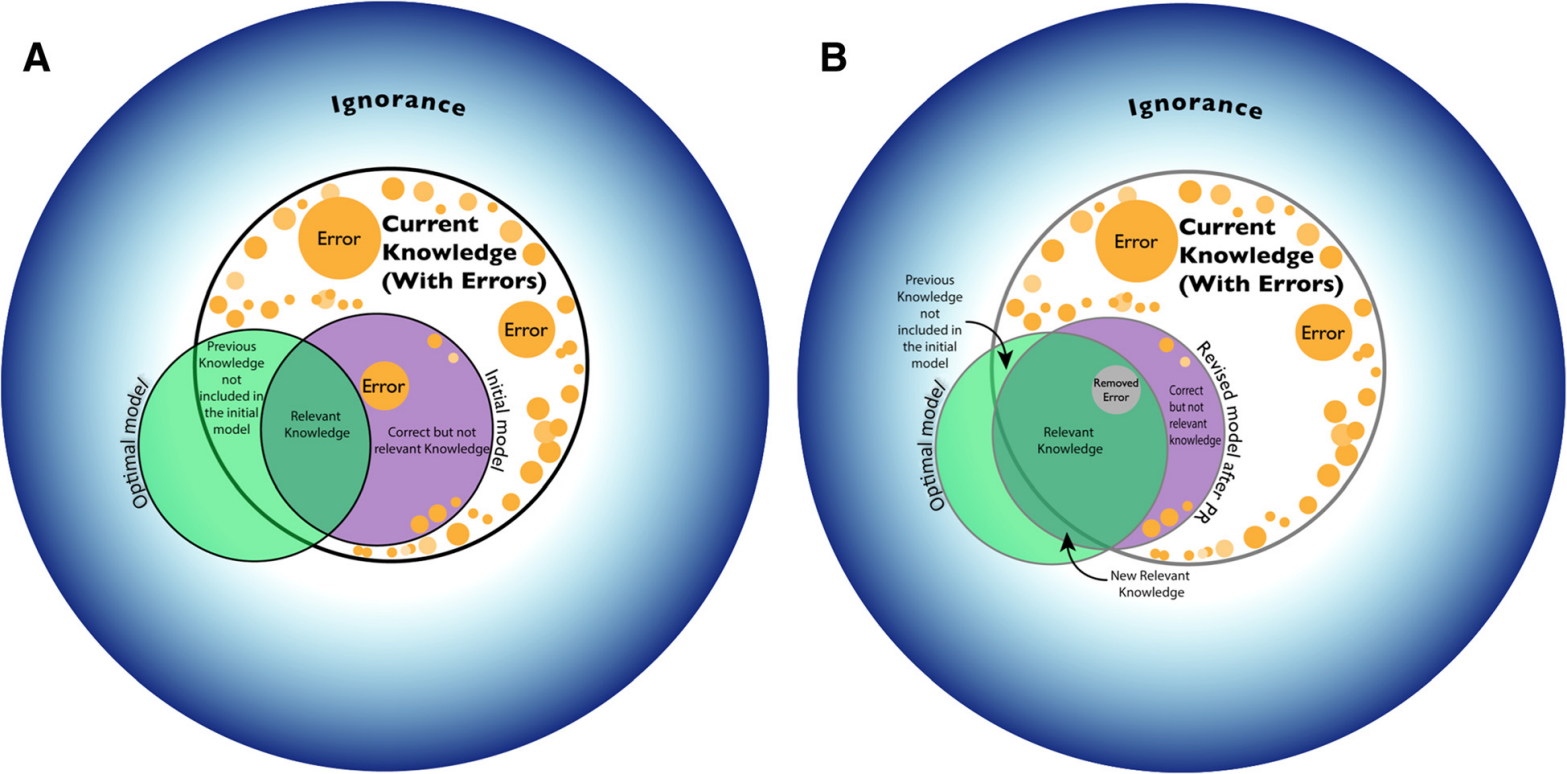
**a** and **b** The use of participatory research to approach the optimal model

Figure 1 illustrates a basic conceptualisation of knowledge in which the set of our beliefs is represented *within* the large circle (white region, labelled ‘Current Knowledge’) and ignorance *outside* the circle (blue region, labelled ‘Ignorance’). The smaller circles (orange region, labelled ‘Error’) represent the part of our beliefs which are in fact false. The remaining space within the large circle represents our beliefs which turn out to be true (or at least sufficiently so). Given this knowledge framework we can only choose a model with a specification (represented by the purple, medium circle and labelled ‘Initial Model’ in Fig. 1a) which falls within the large circle of our beliefs (‘Current Knowledge’) as it is impossible to model something about which we are completely ignorant (Fig. 1a).

Figure 1b illustrates the benefits of best practice use of participatory research. It shows how its employment can lead to a move beyond the constraints of limited, current knowledge towards a more representative and, thus, useful model specification. This is summarised in the figure as an ‘Optimal Model’ (green region). Of course, an Optimal Model is not *omniscience* (total, perfect knowledge), but it is the best representation of reality containing only the necessary and sufficient information to address the questions of interest. While the model in Fig. 1 is highly abstract, it is useful in illustrating the key ways in which participatory approaches can improve model specification:

1. Removing ignorance. Interacting with local communities can provide information that we did not know *a priori*. Examples of this are given in the case studies below.
2. Confirming our *a priori* knowledge. Outputs from participatory research can corroborate our prior understanding.
3. Removing irrelevant information. Participatory research can help to eliminate components of the system that it turns out do not influence the prediction; for example, human movements to market when no vectors of disease have ever been encountered going to market.
4. Including neglected information. Participatory research can remind us of things that we already knew, but had neglected. For example, the theoretical model for Rift Valley fever includes the temporal dynamics of water bodies, which initially was assumed to be driven solely by the environment (seasonal rainfall and evaporation), while anthropogenic activities, although accepted in principle, were not considered of practical relevance. However, participatory research revealed that these patterns can also be driven and altered by irrigation practice during the dry season.
5. Removing erroneous information. Participatory research can correct errors in our thinking; things that we thought were true, but which turn out to be false. For example, modelling spatial movements of goat populations (which may otherwise be an important component of the system), when it turns out that goats do not forage in the area, or investigated source of periodicity in disease transmission when it emerges that the seasonality is an artifact of detection.

We give examples below of the impacts of participatory research on the choice of model structure. Beyond this, we describe the utility of participatory research for identifying the most relevant parameters of the system, identifying the most relevant regime of the system (e.g., temporal stability or otherwise), the feedbacks from mathematical models to guide participatory research and going beyond the so-far described two-way interplay between participatory and mathematical approaches to consider the integration of multiple methods and frameworks.

## Case studies

### Example 1.1 Participatory research as a tool to structure a model: Lassa fever in Sierra Leone

In Sierra Leone, information on livelihoods, lifestyle and movements was collected using participatory techniques, with the mathematical modeller contributing to this fieldwork to enable specific questions to be asked. This allowed the model to be altered, allowing for important influencing factors to be included. For example, it emerged that farmers burn their fields after harvesting. This practice is important because it may drive potentially infected rodent species towards the villages seasonally. Thus, the model structure could be amended to include a periodically varying rate of contact with humans [28].

Additionally, participatory research found that farmers take their threshed rice into barns in their settlements and rodents follow this food source. Also, women mainly cultivate the lowlands after the rice harvest by brushing and clearing emerging weeds and residual rice stalks before burning them. Thereafter, male labour is hired to make mostly raised beds for the cultivation of vegetables. The findings from the participatory research reveal more complex patterns in the contact rate with infected rodents and also with infected humans. Modelling such contact rates with a single numerical value is an oversimplification and the effects of different functional forms for the exposure ought to be assessed [28].

The complex socio-behavioral patterns in contact rates may also help identify characteristics of ‘super spreader’ human disease transmitters who are individuals who can infect a disproportionately large pool of susceptible people [2]. Super-spreading events have been documented for many infectious diseases, but the underlying reasons for super-spreading are not fully understood [29–35]. These probably involve a concomitant range of factors, including physiologic, (e.g. the amount of pathogen excreted, length of the infectious period) social behavior and environmental factors.

There has been some suggestion that incidence of Lassa fever in humans is higher during the dry season, although this view is currently challenged and admissions to Kenema Government Hospital (KGH) appear to occur uniformly during the year (Dr. Donald Grant, personal communication) [36, 37]. Identifying seasonal patterns from the date of admission to hospital [38] is rather challenging due to the limited temporal domain of the data (four years) and a change in policy during the time of collection [39]. Instead, we found that it was more effective to prioritise participatory research to assess whether or not the *apparent* seasonal incidence of Lassa fever in humans is an effect of data collection by, or reporting to, the hospital. Participatory modelling was employed to gauge infrastructure quality (roads are often flooded during the rainy season), economic and social factors (people have limited economic resources in the rainy season), and assess how this affects the reliability of data collection by, or reporting to, the hospital. The outcomes from participatory research indicated the nature of the mathematical approach (seasonal or non-seasonal).

An objective was to estimate the proportion of the burden of Lassa fever in Sierra Leone associated with human-to-human transmission only [2, 40, 41]. The mathematical approach relies on the assumption that the infectious individuals admitted to KGH [38] mix uniformly with susceptible individuals throughout the entire Sierra Leonean population. The rationale behind this is the so-called ‘law of mass action’ [2, 6], a common assumption in epidemiology. Adoption of the law of mass action was supported by previous evidence of large human mobility in Sierra Leone for livelihoods, work and trade, social visits, events [42, 43] and also escaping conflicts [44]. However, it is essential to acknowledge that inputting the actual patterns of mobility and social networking, and hence potential contact patterns, is likely to increase the accuracy of the mathematical approach, and this represents an area where participatory modelling can contribute and is much needed.

Similar integrative efforts were made using further information from fieldwork in Sierra Leone. Focus group discussions and transect walks revealed where different people and rodents go at different times of year. Youths and adults with unrestricted access to uplands cultivate rice in mixed stands at the beginning of the rainy season (May to June) and the owners of lowland fields (mostly male heads of households) cultivate rice in pure stands from the middle of the rainy season (July to August). In the dry season (November to April), the post-rice lowland fields are accessed mainly by women and female youths to cultivate mainly market-oriented vegetables. At this time, resident and migrant youths and adult males instead engaged in mining minerals (diamonds in our case study sites) as well as preparing upland fields for the ensuing rainy season. Moreover, participatory mapping at different times in the farming calendar revealed that some rodent species are confined to the cultivated fields and nearby fallow bushes throughout the cropping season, while others migrate permanently to the settlements after the harvesting of rice in both upland and lowland fields, giving some insight into risks of contact with rodents by different groups. Similarly, Kernéis et al. provided detailed information on prevalence and risk factors of Lassa (e.g. history of collecting, cutting and eating rats) stratified by age, giving precedence for this approach [45]. Based on such information, which indicates non-homogeneous mixing, it is possible to build a more complicated model structure to capture some of these effects. One possible approach is, for example, to adopt a cluster-based inference of the reproduction number by combining the information from participatory research, and/or from Kernéis et al., with age distribution from hospitalised patients at KGH to build a matrix of transmission rates among age groups and between rodents and each age group [45–47].

It was revealed during focus groups and participatory mapping with different gender and age focus groups that rodent species, confined to the cultivated and fallow fields, are hunted for meat by humans using dogs and nets because they are “sweet and oily”. This, is in spite of having being informed of their potentially harmful effect on human health by the KGH’s Lassa Fever Project operating mainly in the case study districts. For those rodent species that migrate into settlements after the rice harvests, the focus group participants indicated that they come into contact with humans through droplets of their faeces and urine from the ceilings of mainly poorly structured houses, mainly occupied by poor people, that contaminate unprotected food and water. It is clearly important, that any modelling approach focusing on exposure and risk factors, needs to consider these parameters and not only official assurances that people know that rodent meat is dangerous.

### Example 1.2 Participatory research as a tool to structure a model: Trypanosomiasis in Zambia and Zimbabwe

An ABM is a class of computational models for simulating the actions and interactions of autonomous agents, both individual and/or collective entities such as organisations or groups, with a view to assessing their effects on the system as a whole [48]. ABMs are particularly suited to integrate with participatory research as both are potentially holistic and both, thus, share a common potential for integration [49].

Participatory research can assist in minimising the error between actual and simulated activities of agents and, thus, lead to a more realistic model. For example, in Zambia in relation to modelling daily activity patterns it was possible through participatory research to capture the types of activities undertaken by the different roles held within households, to capture the range of typical destinations and obtain a *sense* of the frequency and timing of visits (more specification of the model than estimation of parameters). It was then possible to validate the structure of a human movements questionnaire being delivered across the study region, which provided the additional quantitative data required to build the ABM (more estimation of parameters than specification of the model). Thus, participatory research delivered the what, where and why (i.e. qualitative information), whilst the social survey augmented this with the specifics: where, when and how often (i.e. quantification).

Although not used in this way in Zambia, part of the purpose of gathering participatory information can also be to condition human agent movement patterns within an ABM directly; capturing, for example, the time that an agent leaves the household, the direction and speed of their movement, the duration of their stay at their destination and when they return home. Thus, participatory mapping offers an alternative to social survey and diary keeping and, while all can be valid, the meaning and precision of the representation possible with participatory approaches can be important. For example, subtle changes in routes can affect contact probabilities and disease transmission rates in simulations [50].

Participatory research may also provide insights into which parts of daily routines and livelihood activities are the most risky and which people are most at risk, for example, by gender, age group or livelihood [51–53]. Consider that contact networks are key to transmission dynamics. ABMs offer the potential to build a contact network from the bottom up, conditional upon the model specification and using geographical boundary and initial conditions as constraints. At the same time, participatory approaches offer the possibility of targeting this contact information directly (e.g. ask participants where they encounter tsetse and how often). The interplay between these two sources of key information underpinning the transmission system (one conditioned by what is possible given assumptions and the other a direct but uncertain realisation) offers tantalising possibilities for confirmation or rejection of model structure as well as confirmation or rejection of the uncertain information gathered from communities.

In Zimbabwe, participatory mapping in Chitindiva village, dominated by the Korekore people, and Kabidza areas, that house Karanga migrants from south western Zimbabwe, showed that people encroach into forests infested with tsetse according to their ethnicity. Such knowledge will be useful in structuring the ABM model of the area. Similarly, in Zambia, participatory mapping revealed that villagers encountered tsetse in the cotton fields while farming, which was not expected to be a dominant response. This information needs to be integrated with tsetse data and predictive distributions of tsetse abundance which are a key input to the ABM.

In Zimbabwe, one objective was to estimate the prevalence of trypanosomiasis in cattle. Modellers operate under the assumption that trypanosomiasis is a function of animal movement. Through participatory research, it was found that movement of cattle is seasonally-based, and that this results in infected cattle from frontier (forested) areas passing disease to those in established villages long cleared of the fly, allowing for more representative modelling. Moreover, in Zimbabwe, participatory mapping has demonstrated that the wildlife population has changed through time as a result of agricultural intensification and animal poaching that has taken place to satisfy the urban demands for meat following the collapse of commercial agriculture. This information can be used to improve the accuracy of other models.

### Example 1.3 Participatory research as a tool to structure a model: Rift Valley fever in Kenya (RVF)

Participatory studies were used to inform the structure of a mathematical model being developed for RVF in Kenya. Participatory mapping and timelines were used to plot movement patterns of domestic animals between wet and dry season grazing grounds. The timing of such movements was captured, as well as the movement ranges of the various livestock species. The results suggested that cattle move more frequently and across wider spatial ranges than small ruminants, including sheep and goats. Participatory mapping also enabled the research team to identify areas where livestock come into direct or indirect contact with wildlife hosts. In some cases, communities were able to identify wildlife species that are common in these areas.

The above exercise also generated data on practices that increase the risk of RVF exposure in humans; these include taking care of sick animals, and disposal of carcasses and aborted fetuses (RVF causes a large number of abortions in domestic animals). In irrigated areas, both women and men take turns to guard their crops against marauding baboons and other wild birds, especially in late afternoon to early evening. This practice is thought to increase the chances of being bitten by mosquitoes, and hence the risk of exposure to RVF and other vector-borne diseases. These are critical pieces of information that are important in constructing the daily activity patterns of hosts in the model.

For the RVF model, participatory studies contributed to the development of the host module. The ages of cattle and sheep, the main hosts used in the model, were structured into four age classes based on the information obtained from participatory studies. Participatory rural appraisals conducted with the Somali pastoralists in the RVF study site identified the four cattle and sheep age groups as well as the durations that an animal would spend in each age class. Researchers used proportional piling techniques to determine the distribution of hosts by age class. This information was used to evaluate model predictions on host population sizes by age class.

### Example II. Participatory research as a tool to select the most relevant parameters of the system

Stability analysis can benefit from the inclusion of participatory data. In general terms, stability analysis contributes to understanding what happens when a system is perturbed. Common questions in stability analysis are whether or not small population perturbations will dampen out, returning the system to its equilibrium configuration, and if and how variation in the parameter values results in qualitative variation of the solution [54]. Such analysis can be applied, for example, to seasonal systems [55]. Clearly, if something meaningful is to be interpreted from stability analysis we need to know which parameters are more likely to be subjected to variation compared to others. For example, one consortium case study involved studying the ecology of fruit bats in Ghana. In this case, participatory research assisted in identifying the relevant sources of perturbation. More precisely, it was found that bats are an important source of bush meat, and hunting is commonly practised [56]. This translates into a variational increase in the bat mortality rate, resulting in a more meaningful exploration of the space of parameters. The case study of RVF in Kenya is another pertinent example. The disease is largely associated with water bodies, which are breeding sites for the mosquitoes carrying the infection. Usually rainfall data are used as proxies for water bodies, however from participatory analysis it emerged that irrigation patterns can also play an important role in creating additional, temporarily varying breeding sites, with patterns potentially different from the rainfall cycle. Therefore the model for stability analysis of the system was amended to allow these additional patterns [57].

In Kenya, participatory methods such as relative incidence scoring were used to compare RVF incidences and case fatality rates among different livestock species and age classes. In this case, pastoral communities were involved in games and exercises that involved clustering livestock into different species and age classes, using counters such as pebbles or seeds. After this, pastoralists were asked to use past experiences of RVF to indicate their perceptions of the relative proportion of animals that would be affected (in terms of incidence, mortality or abortion) in each group. Data obtained from these exercises were used to weight case fatality and abortion rates, especially when age and species specific parameters were not available.

### Example III. Participatory research as a tool to identify the most relevant regime of the system

Many theoretical approaches, e.g. stability analysis, emphasise equilibrium states. Participatory modelling can assist in determining whether or not the system has reached such an equilibrium configuration, identifying the possible causes leading to a disruption of the equilibrium. It can also direct the mathematical approach towards the relevant regime, that is, a transient regime rather than equilibrium. For instance, in recent years cashew nuts have become an important industry in Ghana [58]. According to preliminary outcomes from participatory modelling, the proliferation of large cashew nut plantations is currently affecting the dispersal patterns of fruit bats, a reservoir of many viruses including Ebola, rabies and Nipah [59]. Related use of pesticides is also increasing with a potential effect on the survival of bats. In certain locations, the hunting patterns are also subjected to change, such as in the area around Tano sacred grove, the location of one of the largest roosts in Ghana, where the local chief has granted permission for hunting. All this information, emerging from interaction with the local community, suggests that in many cases the ecological system of bats is far from in an equilibrium situation.

In the Lassa fever case study in Sierra Leone, it was found through focus group discussions that rodents once inhabited forest lands, but as their habitat is being disturbed through farming, coupled with shortened fallow periods, they are now confined to less than five-year-old fallow farmlands. This information can be used to predict the equilibrium states of rodents in association with changes in land-use partners. The precision of the model using local knowledge could be improved when triangulated with the results from rodent trappings and monitoring of the species associated with land-use changes by both the epidemiological, environment and land-use teams in the project.

### Example IV. Feedback from modelling efforts as a tool to improve the design of participatory research and provide new areas of interest to study

The examples above reveal the potential information flow from participatory approaches to mathematical modelling. Here, we present examples showing how outcomes from mathematical modelling can indicate further areas to be explored using participatory approaches.

One of the early findings of our theoretical approach was the relatively high impact of human-to-human transmission of Lassa fever. This might be associated with the long persistence of viruria, even during the recovery period, explaining the long time for shedding of the disease, especially in rural settlements where sanitary facilities are limited [60, 61]. Participatory modelling can use this information to explore new areas, for example effectively assessing the variety of practices and settings in which people come into contact with each other’s bodily fluids, and their approaches to hygiene. It was revealed through focus group discussions with different gender and age groups that rodents bite the limbs of inhabitants of dwelling places while they are asleep. This increases the chances of household members coming into contact with each other’s body fluids, particularly in poorly structured dwellings in both urban and rural locations in the case study sites. Participatory modelling could also be used to elucidate if and how caring behaviour and the relative perceptions of risk change patterns of behaviour.

In Zimbabwe, data being gathered for an ABM have produced possible directions for participatory research. A precise record of human and animal movements obtained by questionnaire and simulation of human movements based on realistic constraints is helping to direct further questions. The questions in the survey of *who* actually moves rather than whether there is movement, and where people go and at what times, has directed participatory research to look at the politics of such movement.

### Example V. Diversity of modelling approaches challenges the conclusions of other types of modelling

The above examples have illustrated the potential benefits of one-way interactions between participatory and mathematical modelling approaches. However, the greater challenge is to integrate a wide range of different methodological approaches (which, in the case of our consortium, means five approaches).

Reliance on a single modelling approach is always risky as no model can claim to capture everything; reality is too complex to model in full. Different models highlight different issues and are based on different assumptions, world views and sources of information, leading to different conclusions about disease risk and the appropriate actions and policy decisions to take [6]. This makes choosing one approach over another problematic. Interdisciplinary working can address these issues, embracing multiple sources of evidence [62].

For example, in contrast with numerical datasets and various types of mathematical modelling, conceptual characterisations derived from ethnographic and participatory research offer contrasting views ‘from the ground’, which may question dominant policy actions [6], including feedback from local communities on the findings coming from traditional research, as well as the benefits of participatory research itself. This can lead to an enriched interpretation of research findings, integrating different disciplinary perspectives, and a wider-ranging translation of research. It can also mean that there is more opportunity for wider dissemination among many different audiences and that the integrated models will therefore be potentially more useful in practice and policy.

This is important to note because, at times, attention to local people in zoonotic disease research has come only when researchers’ non-participatory perspectives have centred around people, for example, when humans have become vectors themselves of the disease or when human impact on wildlife and the environment is being considered. In Zimbabwe, for example, people are frequently condemned for encroaching into wilderness areas, where tsetse abound, and importing trypanosomiasis back into mainstream society. Participatory approaches can provide local assessment and a rationale for local practices appearing to be a driver of disease, especially in areas where detailed data sources are often unavailable. Without local people’s input, models may provide predictions and explanations based only on a certain outsider account of actuality.

## Conclusions

This paper argues that reality is too complex to be modelled by one modelling approach from one discipline; an integrated approach can increase the accuracy of models and understanding. Crossing professional, disciplinary and institutional boundaries, challenging as this may be, to work in a more integrated fashion can have great benefits [62–65]. The paper has focused on the many benefits of the use of participatory approaches to lead to more realistic mathematical models to assist with policy decisions aimed at reducing disease and benefiting local people.

This paper has also given examples that have demonstrated how participatory research can be guided by other methods, and the integration of multiple methods and frameworks. It must however be realised that participatory research is not a ‘cheap alternative’ to collecting quantitative data [12, 66]. The value of participatory research is rather to highlight beliefs, behaviours and practices that are unfounded scientifically, thus, showing that its value can lie in its combination with other forms of data.

The types of possible integrative framework, including scenario planning, can be done with key stakeholders including local communities [67, 68]. For this to be successful there needs to exist an openness to new integrative approaches, and respect and collaboration among disciplines who may speak different languages [62, 69]. This, above all, is key to the success of interdisciplinary integration.

## Additional file

Additional file 1: Multilingual abstracts in the six official working languages of the United Nations. (PDF 351 kb)

## Abbreviations

ABMs: Agent- based models
CAMPFIRE: The Communal Areas Management Programme for Indigenous Resources
KGH: Kenema Government Hospital
RVF: Rift Valley Fever
SLESRC: Sierra Leone Ethics and Scientific Review Committee
