# Supplementary material for: Moving interdisciplinary science forward: integrating participatory modelling with mathematical modelling of zoonotic disease in Africa
Source: Infect Dis Poverty. 2016 Feb 25;5:17. doi: 10.1186/s40249-016-0110-4 (PMC4766706; doi:10.1186/s40249-016-0110-4)
Supplement: Additional file 1: — Multilingual abstracts in the six official working languages of the United Nations. (PDF 351 kb) [file 40249_2016_110_MOESM1_ESM.pdf]

Translation of the abstract into the six official working languages of the United Nations

إحراز تقدم في العلوم متعددة التخصصات: دمج النمذجة التشاركية مع النمذجة الرياضية للأمراض حيوانية المنشأ في أفريقيا

كاثرين جرانت، چيوڤاني لو إياكونو، ڤوپنيو دزينجيراي، برنار بت، توماس ر. أ. وينيبا، پيتر م. أتكينسون

ملخص

يبين هذا الاستعراض فوائد استخدام مناهج متعددة لتحسين تصميم النماذج وتسهيل البحوث متعددة التخصصات في الأمراض المعدية، وكذلك يبين ويقترح أمثلة عملية ذات تكامل فعال. ويهتم بصورة خاصة بفوائد استخدام البحث التشاركي بالتزامن مع أساليب النمذجة التقليدية التي قد يؤدي لتحسين بحوث ومراقبة وإدارة الأمراض. ويمكن أن تؤدي المناهج المتكاملة إلى نماذج رياضية أكثر واقعية وهذا بدوره يمكن أن يساعد في اتخاذ القرارات السياسية التي تقلل من المرض وتفيد السكان المحليين. يتأثر ظهور الأمراض ، ومخاطرها، وانتشارها ومكافحتها بالعديد من العوامل البيولوجية المادية والبيئية والاجتماعية والاقتصادية المعقدة التي تشمل تغير المناخ والبيئة، والتغير في استعمالات الأرض ، والتغيرات في السكان وسلوك الأشخاص. وضعت قاعدة الأدلة لهذا الاستعراض الاستطلاعي استنادا إلى عمل جماعي، بهدف إدماج مناهج النمذجة المستخدمة تقليديا في علم الأوبئة والبحوث البيئية والتنمية. نعرض ما مجموعه خمسة أمثلة من آثار البحوث التشاركية على اختيار الهيكل النموذجي. توصف فائدة البحوث التشاركية في المثال 1، محددا المعايير الأكثر ملاءمة للنظام. المثال 2 يركز على تحديد الأسلوب الأكثر ملاءمة لنظام العمل (على سبيل المثال، الاستقرار الزماني أو غير ذلك)، المثال 3 يدرس التعليقات على النماذج الرياضية لتوجيه البحوث التشاركية والمثال 4 يتجاوز التفاعل في اتجاهين بين المناهج التشاركية والرياضية الموصوف بالفعل من خلال دمج أساليب وأطر متعددة. يصف هذا الاستعراض الاستطلاعي أمثلة على أفضل الممارسات في استخدام الأساليب التشاركية، موضحا قدرتها على التغلب على العقبات التخصصية وتعزيز التعاون متعدد التخصصات، وذلك بهدف جعل النماذج وتوقعاتها أكثر فائدة لاتخاذ القرارات وصياغة السياسات.

Translated from English version into Arabic by Mahmoud Sami, through

TRANSLATORS
WITHOUT BORDERS

推进交叉学科发展：整合非洲人兽共患传染病参与式建模和数学建模

Catherine Grant, Giovanni Lo Iacono, Vupenyu Dzingirai, Bernard Bett, Thomas R. A. Winnebah and Peter M. Atkinson

摘要

本文概述了使用多种方法进行传染病研究的优势，如完善模型设计和促进多学科研究，并展示和提出了有效整合的实例。特别是在促进疾病研究、控制和管理方面，整合参与式研究与传统建模方法的优势尤其明显。综合的方法能增强数学建模的实际操作性，反过来又可协助政策的制定，从而减少疾病，造福当地人民。疾病的出现、风险、传播和控制受到许多复杂的生物物理、环境和社会经济等因素的影响。这些因素包括气候和环境变化，土地用途转换，人口和人类行为的改变。本堪域综述的实证基础来源于以整合常用于流行病学、生态学和发展研究的方法为宗旨的联盟工作。本文共用五个实例介绍参与式建模对选择模型结构的影响。实例一描述了参与式建模的实用性，确定了该系统的最相关参数；实例二的关注点在于该系统的最相关状态（如时间稳定性或其他方式）；实例三分析数学模型的反馈以指导参与式建模；实例四阐述多种方法和框架的综合运用较之于当前描述的参与式建模和数学建模两种方法联合使用的优越性。本堪域综述描述了参与式建模运用的最佳范例，说明其在跨越学科障碍，促进多学科整合的潜在可行性，提高建模及其预测在政策制定和实施中的作用。

Translated from English version into Chinese by Chen Jin, edited by Yang Pin, through

CHINESE CENTER FOR DISEASE CONTROL AND PREVENTION
NATIONAL INSTITUTE OF PARASITIC DISEASES

Avancement de la science interdisciplinaire : intégrer la modélisation participative à la modélisation mathématique des zoonoses en Afrique

Catherine Grant, Giovanni Lo Iacono, Vupenyu Dzingirai, Bernard Bett, Thomas R. A. Winnebah et Peter M. Atkinson

Résumé

Cette étude présente les nombreux avantages liés à l'utilisation d'approches multiples pour améliorer la conception de modèles et favoriser la recherche multidisciplinaire sur les maladies infectieuses tout en proposant des exemples pratiques d'intégration efficace. Elle s'attarde plus précisément sur les avantages de la recherche participative utilisée en conjonction avec les méthodes de modélisation traditionnelles pour améliorer éventuellement la recherche, le contrôle et la gestion des maladies. Des approches intégrées peuvent conduire à des modèles mathématiques plus réalistes qui, à leur tour, peuvent aider à prendre des décisions politiques qui réduisent les maladies et profitent aux populations locales. De nombreux facteurs biophysiques, environnementaux et socioéconomiques affectent l'apparition, le risque, la propagation ou le contrôle des maladies. Ces facteurs comprennent le réchauffement climatique et les changements environnementaux, les variations de l'utilisation des sols, les changements démographiques et le comportement des gens. La base factuelle de cette étude de portée provient du travail d'un consortium, dans le but d'intégrer les méthodes de modélisation traditionnellement employées en recherche épidémiologique, écologique et de développement. Le document présente cinq exemples des effets de la recherche participative sur le choix de la structure des modèles. L'Exemple 1 montre l'utilité de la recherche participative en identifiant les paramètres les plus pertinents du système. L'Exemple 2 cherche à identifier le régime le plus pertinent du système (p. ex. stabilité temporelle ou autre), l'Exemple 3 étudie les rétroactions des modèles mathématiques pour guider la recherche participative et l'Exemple 4 va au-delà de l'interaction bilatérale entre les approches participatives et mathématiques, décrite jusqu'ici, en y intégrant des méthodes et des cadres multiples. Cette étude de portée donne des exemples des meilleures pratiques liées à l'emploi des méthodes participatives, illustrant leur capacité à déjouer les barrières disciplinaires et à promouvoir la collaboration multidisciplinaire de manière à créer des modèles prévisionnels plus utiles à la prise de décision et à l'élaboration de politiques.

Translated from English version into French by Myriam Grandchamp, through

TRANSLATORS
WITHOUT BORDERS

Интеграция партисипативного моделирования с математическим моделированием зооноза в Африке — продвижение научной деятельности междисциплинарного характера

Catherine Grant, Giovanni Lo Iacono, Vupenyu Dzingirai, Bernard Bett, Thomas R. A. Winnebah and Peter M. Atkinson

Аннотация
В обзоре приведены преимущества применения нескольких подходов для улучшения конструкции модели и облегчения междисциплинарного исследования в области инфекционных заболеваний, а также показаны и предложены практические примеры эффективной интеграции. В частности, речь идет о преимуществах использования партисипативных исследований в сочетании с традиционными методами моделирования в целях потенциального улучшения исследования, контроля и лечения заболевания. Интегрированные подходы могут натолкнуть на более реалистичные математические модели, которые, в свою очередь, могут помочь в принятии политических решений, что снизит заболеваемость и принесет пользу местному населению. Возникновение, риск, распространение и контроль заболеваний зависит от многих сложных биофизических, экологических и социально-экономических факторов, таких как изменение климата и условий окружающей среды, изменения в области землепользовании, изменения численности населения и общественного поведения. Доказательная база для данного обзорного анализа является результатом совместной работы по интеграции подходов моделирования, традиционно используемых в эпидемиологических, экологических исследованиях и исследованиях в области развития. В общей сложности представлено пять примеров влияния партисипативных исследований на выбор структуры модели. Польза партисипативных исследований описана в примере 1, где определяются наиболее значимые параметры системы. Пример 2 сфокусирован на выявлении наиболее значимого режима работы системы (временная стабильность и т. д.). В примере 3 рассматриваются обратные связи с математическими моделями для ведения партисипативного исследования. Пример 4, выходя далеко за рамки, описывает двухстороннее взаимодействие между партисипативными и математическими подходами при интеграции множества методов и механизмов. Данный обзорный анализ описывает примеры передовой практики применения партисипативных методов, иллюстрируя их потенциал в области преодоления дисциплинарных препятствий и продвижения междисциплинарного сотрудничества с целью создания более пригодных для принятия решений и выработки политики моделей и прогнозов.

Translated from English version into Russian by Anna Romanenko, through

TRANSLATORS
WITHOUT BORDERS

Avances en la ciencia interdisciplinaria: integración del modelado participativo y el matemático en las enfermedades zoonóticas de África

Catherine Grant, Giovanni Lo Iacono, VupenyuDzingirai, Bernard Bett, Thomas R. A. Winnebah y Peter M. Atkinson

Resumen

Este análisis resume los beneficios de utilizar múltiples enfoques para mejorar el diseño de modelos y facilitar la investigación multidisciplinaria de las enfermedades infecciosas, así como también para mostrar y proponer ejemplos prácticos de una integración efectiva. En particular, explora los beneficios de usar investigación participativa en conjunto con métodos de modelado tradicionales para mejorar potencialmente la investigación, el control y el manejo de las enfermedades. Los enfoques integrados pueden llevar a modelos matemáticos más realistas que, a su vez, pueden asistir en la toma de decisiones en cuanto a políticas que reduzcan las enfermedades y beneficien a las poblaciones locales. La emergencia, el riesgo, la diseminación y el control de las enfermedades se ven afectados por muchos factores biofísicos, ambientales y socioeconómicos complejos, que incluyen el cambio climático y ambiental, la variación en el uso del suelo, los cambios poblacionales y el comportamiento de los individuos. La evidencia base para este análisis de alcance proviene del trabajo de un consorcio con el fin de integrar los enfoques de los modelos utilizados tradicionalmente en la investigación epidemiológica, ecológica y de desarrollo. Se presenta un total de cinco ejemplos del impacto de la investigación participativa en la elección de la estructura modelo. La utilidad de la investigación participativa se describe en el ejemplo 1, identificando los parámetros más relevantes del sistema. El ejemplo 2 apunta a la identificación del régimen más relevante del sistema (por ejemplo, la estabilidad temporal o de otro tipo), el ejemplo 3 examina las devoluciones de modelos matemáticos para guiar la investigación participativa y el ejemplo 4 va más allá de la interacción bilateral hasta entonces descrita entre los enfoques participativos y matemáticos por integración de múltiples métodos y marcos. Este análisis de alcance describe ejemplos de buenas prácticas en el uso de métodos participativos, ilustrando su potencial para superar obstáculos disciplinarios y promover la colaboración multidisciplinaria con el objetivo de producir modelos y predicciones más útiles para la toma de decisiones y la formulación de políticas.

Translated from English version into Spanish by Aldana Gómez Ríos, through

TRANSLATORS
WITHOUT BORDERS
